# Development and validation of an LC-MS/MS methodology for the quantification of thyroid hormones in dko MCT8/OATP1C1 mouse brain

**DOI:** 10.1016/j.jpba.2022.115038

**Published:** 2022-09-10

**Authors:** Meri De Angelis, Gandhari Maity-Kumar, Sonja C. Schriever, Elena V. Kozlova, Timo D. Müller, Paul T. Pfluger, Margarita C. Curras-Collazo, Karl-Werner Schramm

**Affiliations:** a Helmholtz Zentrum München-German Research Center for Environmental Health (GmbH), Molecular EXposomics, Ingolstädter Landstr. 1, Neuherberg, Germany; b Institute for Diabetes and Obesity, Helmholtz Diabetes Center at Helmholtz Zentrum München, Neuherberg, Germany; c German Center for Diabetes Research (DZD), Neuherberg, Germany; d Research Unit Neurobiology of Diabetes, Helmholtz Zentrum München, Neuherberg, Germany; e Department of Molecular, Cell and Systems Biology, University of California, Riverside, USA; f TUM School of Medicine, Neurobiology of Diabetes, Technical University Munich, Germany; g Department für Biowissenschaftliche Grundlagen, Technische Universitat München, Weihenstephaner Steig 23, Freising, Germany

**Keywords:** dko MCT8/OATP1C1 mouse brain, L-thyroxine (T4), 3,3,5-triiodo-L-thyronine (T3), 3,3,5-triiodo-L-thyronine (rT3), 3,3-diiodo-L-thyronine (3,3-T2), Liquid chromatography-mass spectrometry

## Abstract

The Allan-Herndon Dudley Syndrome (AHDS) is a rare disease caused by the progressive loss of monocarboxylate transporter 8 (MCT8). In patients with AHDS, the absence of MCT8 impairs transport of thyroid hormones (TH) through the blood brain barrier, leading to a central state of TH deficiency. In mice, the AHDS is mimicked by simultaneous deletion of the TH transporters MCT8 and the solute carrier organic anion transporter family member 1c1 (OATP1C1). To support preclinical mouse studies, an analytical methodology was developed and successfully applied for quantifying selected thyroid hormones in mouse whole brain and in specific regions using liquid chromatography tandem mass-spectrometry (LC-MS/MS). An important requirement for the methodology was its high sensitivity since a very low concentration of THs was expected in MCT8/OATP1C1 double-knockout (dko) mouse brain. Seven THs were targeted: L-thyroxine (T4), 3,3,5-triiodo-L-thyronine-thy-ronine (T3), 3,3’,5’-triiodo-L-thyronine-thyronine (rT3), 3,3-diiodo-L-thyronine (3,3’-T2, T2), 3,5-diiodo-L-thyro-nine (rT2, 3,5-T2), 3-iodo-L-thyronine (T1), 3-iodothyronamine (T1AM). Isotope dilution liquid chromatography triple-quadrupole mass spectrometry methodology was applied for detection and quantification. The method was validated in wild-type animals for mouse whole brain and for five different brain regions (hypothalamus, hippocampus, prefrontal cortex, brainstem and cortex). Instrumental calibration curves ranged from 0.35 to 150 pg/μL with good linearity (r^2^ >0.996). The limit of quantification was from 0.08 to 0.6 pg/mg, with an intra- and inter-day precision of 4.2−14.02% and 0.4−17.9% respectively, and accuracies between 84.9% and 114.8% when the methodology was validated for the whole brain. In smaller, distinct brain regions, intra- and inter-day precision were 0.6−20.7% and 2.5−15.6% respectively, and accuracies were 80.2−128.6%. The new methodology was highly sensitive and allowed for the following quantification in wild-type mice: (i) for the first time, four distinct thyroid hormones (T4, T3, rT3 and 3,3’-T2) in only approximately 100 mg of mouse brain were detected; (ii) the quantification of T4 and T3 for the first time in distinct mouse brain regions were reported. Further, application of our method to MCT8/OATP1C1 dko mice revealed the expected, relative lack of T3 and T4 uptake into the brain, and confirmed the utility of our analytical method to study TH transport across the blood brain barrier in a preclinical model of central TH deficiency.

## 1 Introduction

**Fig. 1:**
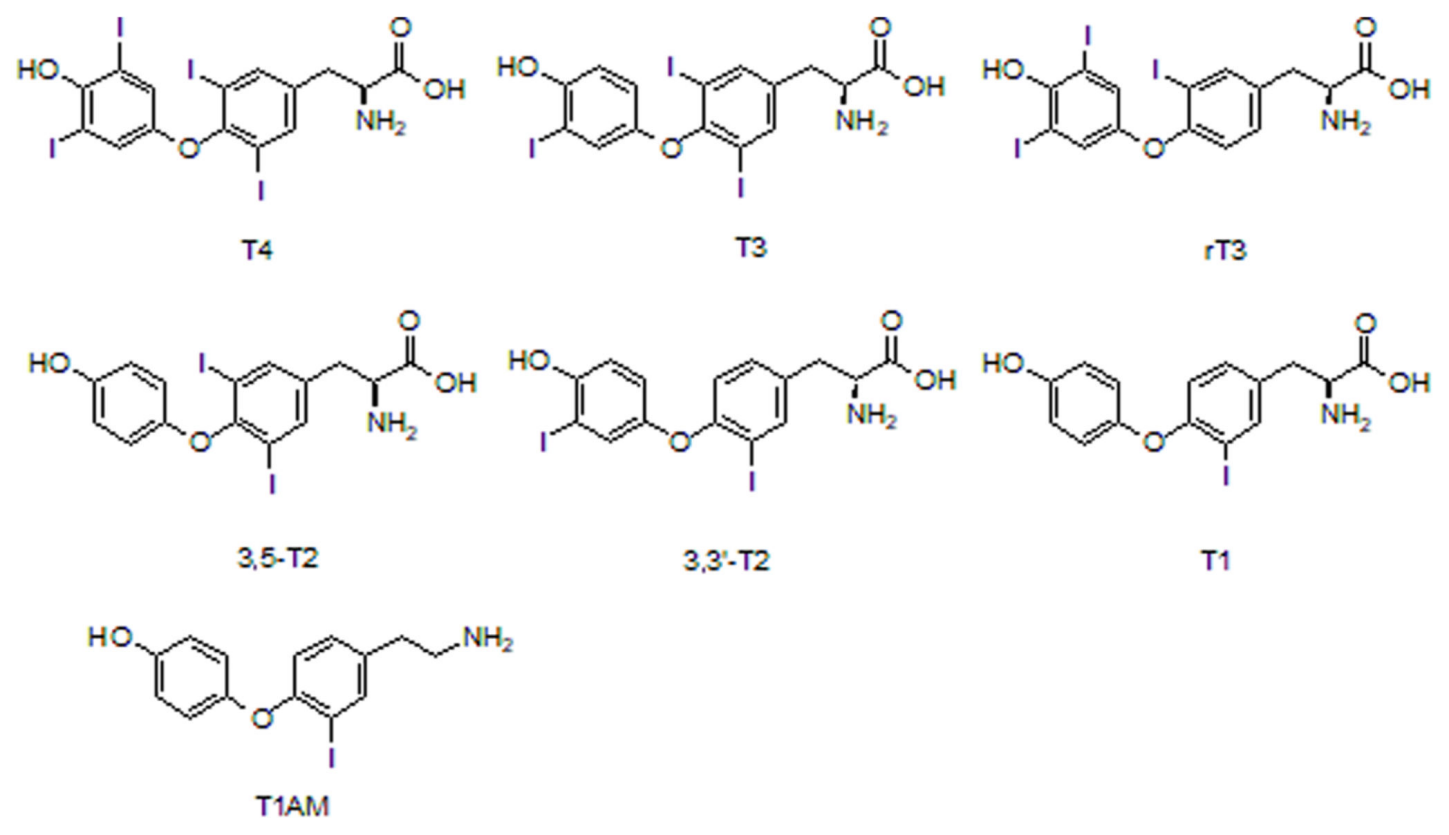
Molecular structure of the thyroid hormones under investigation.

Thyroid hormones (TH) are a class of tyrosine-based hormones (Fig. 1). They are synthesized by the thyroid gland as part of the hypothalamic-pituitary-thyroid (HPT) axis cascade and can affect many vital physiological functions. However, L-thyroxine (T4), the main hormone produced by the thyroid gland has only a minimal effect on stimulating body metabolism. T4 must be converted into 3,3’,5-triio-dothyronine (T3), the most active form, by intracellular removal of one iodine atom in order to exert its biological effects. In turn, T3 and T4 are inactivated into 3,3’-diiodo-L-thyronine (3,3T2) and 3,3’,5’-triiodothy-ronine (reverse T3 or rT3), respectively. Further deiodination events can generate lower iodinated analogues such as, 3,5-diiodo-L-thyronine (3,5-T2, reverse T2 or rT2) or monoiodothyronine (T1) [1,2]. However, thyroid hormones are also decarboxylated or deaminated giving rise to other metabolites. Among those, 3-monoiodothyronamine (T1AM), first detected in rat brain [3], has recently garnered attention due to its rapid and profound pharmacological effects on body temperature, heart rate and metabolism [4–7]. However, its physiological role as endogenous metabolite remains to be fully understood [7,8].

Among all the physiological processes controlled by thyroid hormones, one of the most important is regulation of brain development and function, highlighting the importance of an adequate delivery mechanism for TH across the blood brain barrier (BBB). In the Allan-Herndon Dudley Syndrome (AHDS), a rare disease caused by the loss of monocarboxylate transporter 8 (MCT8), this transport of TH across the blood−brain barrier is deficient. Consequently, patients with AHDS show severe intellectual and motor disability [9,10]. They are typically unable to speak, and often need assistance with eating, walking and sitting upright [11]. In mice, the AHDS is mimicked by simultaneous deletion of the thyroid hormone transporters MCT8 and the organic anion transporter1 C1 (OATP1C1). MCT8/OATP1C1 dko mice show impaired T3/T4 transport across the BBB with central TH deficiency [12]. Like AHDS patients, MCT8/OATP1C1 dko mice show locomotor deficits and peripheral hyperthyroidism, making these mice a good model to evaluate novel pharmacological treatments for AHDS.

To support our preclinical mouse studies, an analytical methodology aimed at the quantification of THs in mouse brain was needed. An important requirement for the methodology was high sensitivity since a very low concentration of THs was expected in MCT8/OATP1C1 dko mouse brain [12]. In the past, initial attempts to measure TH concentrations in rodent brain were achieved using radio immunoassay (RIA). With this methodology, it was possible to quantify T3 and T4 in rat brain and also in single rat brain regions [13–15]. Some authors also reported the quantification of up to seven different TH metabolites in different rat brain regions [16]. Due to the high sensitivity of this technique, the first studies conducted in MCT8/OATP1C1 dko mice used this methodology to measure the low T3 and T4 brain concentration [12]. However, not all laboratories are able to work with radioactive material and alternative methodologies are needed. Over the last two decades, many analytical methods have been developed that use liquid-chromatography mass spectrometry (LC-MS) or tandem mass spectrometry (LC-MS/MS) to measure THs due to the high sensitivity and selectivity of this technique. Using mass spectrometry, it has been possible to quantify TH levels in many different matrices (serum, tissues, cells, hair, milk, etc.) both in animal and human samples [17–28]. Among them, some papers described the application of such technique also in rodent brain. Kunisue et al. were able to quantify T3 and T4 in 500 mg of rat brain, while Saba et al. were able to further quantify T1AM using similar tissue quantities [22,29]. The same methodology described by Saba and co-workers was employed, some years later, for measuring T4, T3, and T1AM on mouse brain [30]. Of note, despite the relatively high quantity of rodent brain tissue (about 500 mg), only a few thyroid hormones were measured in those studies. Moreover, none reported on the quantification of THs in specific brain regions, which are generally limited in quantity in mouse brain. Because of the division of labor in the brain by region, such data are of great interest as they allow a better understanding of the role of THs in the respective areas. For example, how the regional specificity of TH distribution in the brain might correlate with distinct cognitive functions still remains largely elusive.

In our group, we have recently developed an LC-MS methodology for the quantification of thyroid hormones (T4, T3, rT3, 3,3’-T2, 3,5-T2, T1 and T1AM) in different animal and human tissues using isotope dilution (^13^C_6_-T4, ^13^C_6_-T3, ^13^C_6_-rT3, ^13^C_6_-3,3’-T2 and ^13^C_6_-T1AM). With our method we were able to detect and quantify T4, T3 and, in some tissues, also rT3 and 3,3’-T2 [24,25]. Thus, we decided to apply our protocol also for the extraction and detection of thyroid hormones from mouse brain. The methodology was validated using between 70 and 100 mg (out of 350−400 mg total wet brain weight) of the entire brain homogenate obtained from C57BL/6 mice. When the procedure was applied to approximately 100 mg of mouse brain, T4, T3, and for the first time rT3 and 3,3’-T2 were quantified. To broaden the applicability of our protocol, the methodology was further tested for the measurement of THs in distinct brain regions. To test the feasibility of our methodology, we measured the intra-day and inter-day accuracy and precision using about 15 mg of brain tissue. After this partial validation, the procedure was employed for the detection of THs in the hypothalamus, hippocampus, prefrontal cortex, brainstem and cortex of C57BL/6 mice. In all samples tested, T4 and T3 were easily detectable and quantifiable. No other metabolites were quantified, although rT3 and 3,3’-T2 were both detected in all the prefrontal cortex samples. To the best of our knowledge, this is the first report, describing the possibility of using mass spectrometry to measure the main thyroid hormones, T4 and T3, in distinct mouse brain regions. This proves the high sensitivity of our methodology which was finally applied to the quantification of THs in dko MCT8/OATP1C1 mouse brain.

## 2 Material and methods

### 2.1 Chemicals and reagents

T4, T3, rT3, 3,5-T2, T1, T1AM, ^13^C_6_-T3, ^13^C_6_-T4, ^13^C_6_-rT3 were obtained from Sigma-Aldrich (St. Louis, MO, USA). 3,3’-T2 and ^13^C_6_-3,3’-T2 were obtained from Santa Cruz Biotechnology (Dallas, Texas, USA). ^13^C_6_-T1AM was purchased from Toronto Research Chemicals (North York, Ontario, Canada). All other reagents were purchased from Sigma-Aldrich (St. Louis, MO, USA). Solvents were purchased from Promochem (Wesel, Germany) and water from Merck Millipore (Darmstadt, Germany). All reagents were ACS or LC-MS grade. Bond Elut Plexa PCX cartridges (60 mg, 3 mL) were purchased from Agilent Technologies (Santa Clara, CA, USA).

### 2.2 Preparation of standard solutions

The standard solutions, as well as the quantification standards, were prepared as previously described [25]. In brief: a stock solution of 100 ng/μL for 3,3’-T2, and of 50 ng/μL for 3,5-T2, and T1 was prepared by dissolving the desired thyroid hormone in 0.1 N NH_4_OH in CH_3_OH (the same solvent in which the commercially available thyroid hormone solutions are prepared). While, a stock solution of 100 ng/μL of T1AM and ^13^C_6_-T1AM was prepared in pure CH_3_OH. All other standards were commercially available in solution (0.1 N NH_4_OH in CH_3_OH) at a concentration of 100 ng/μL. Those bulk solutions were stored at −20 °C. Calibration standard solutions (0.35−150 pg/μL of each analyte and 10 pg/μL of the internal standards) were prepared using 0.1% formic acid in H_2_O:CH_3_CN (8:2, v/v) and they were stored at 4 °C.

### 2.3 Instrumentation

Analysis of THs was performed with Agilent 1290 Infinity II LC system interfaced with an Agilent 6470 triple quadrupole tandem mass spectrometer. The flow rate was set at 0.3 mL/min, the column used was a Zorbax Eclipse Plus C18 (2.1 × 50mm, 1.8 μM) with the column temperature fixed at 40 °C. The injection volume was always 20 μL. The mobile phases were water (A) and acetonitrile (B) each containing 0.1% formic acid (v/v). Gradient elution was performed according to the following HPLC gradient: 0−3 min 5% B; from 3 to 4 min ramped linearly to 30% B; from 4 to 5.5 min gradually increased to 38% B; 5.5−6.5 held for 1 min at the same condition, followed by an increase to 40% B until minute 7.5. These conditions were kept until minute 9. Finally, B increased to 100% for 3 min and kept for an additional minute. The program returned to initial conditions in 0.2 min followed by 3.8 min for re-equilibration before the next injection. Analytes were detected using an electrospray ionization source (ESI) in positive mode. The tandem mass spectrometer was operated under multiple reaction monitoring mode (MRM). The MS/MS transitions (*m/z*), the fragmentation voltage (FV), the collision energy (CE), the cell accelerator voltage (CAV) and the dwell time for the transitions are reported in Table S1 of the supplementary material. The other mass spectrometric parameters were set as follow: gas temperature 125 °C; gas flow 6 L per min; nebulizer pressure 55 psi; capillary voltage 5500 V.

### 2.4 Sample preparation for TH analysis

For the whole brain, samples were pestled at − 200 °C to homogenize them and to obtain a powder from which about 100 mg of brain were used for the extraction of THs. This powder tend to melt easily if not kept frozen. To reduce this complication during weighing, the entire brain powder contained in an Eppendorf vial was kept in liquid nitrogen together with the spatula needed to take the sample. With the “frozen” spatula, the power was collected and immediately weighted in a new 1.5 mL Eppendorf vial. Following this process samples can only melt after being weighted, however they were always processed immediately afterwards. To this material, inserted into a 1.5 mL Eppendorf vial, pure methanol (300 μL) was added. The sample was homogenized by ultrasonication (Bandelin Electronics, Berlin, Germany) under cooling for 30 s. After homogenization 60 μL of internal standards (10 pg/μL) and 0.6 mL of chloroform were added. The mixture was vortexed for 10 s and then centrifuged (Multifuge 3 S/3S-R, Thermo Scientific, Walth-mann, USA) at 3000 g for 10 min. The supernatant was then collected and the extraction procedure repeated one more time using a 2:1 mixture of chloroform and methanol. The organic extracts were transferred into a 12 mL tube for back-extraction of the iodothyronine with 800 μL of 0.05% CaCl_2_ in water. The mixture was centrifuged again and the supernatant collected. The extraction procedure was repeated by adding 800 μL of methanol and 800 μL of 0.05% CaCl_2_ in water, respectively. To the pooled aqueous phase, 2 × 2 mL of n-hexane: chloroform (9:1, v/v) was added, the mixture was vortexed for 10 s and the upper phase removed and discarded. To the lower phase, 1 mL of pure water was added followed by the addition of H_3_PO_4_ (1%, v/v). After vortexing, the solution was transferred to a Bond Elut Plexa PCX cartridge that was preconditioned with 2-mL of CH3OH and 2-mL of H_2_O. Thereafter, the cartridge was washed with 2-mL of 2% formic acid in water and 2-mL of CH3OH:CH3CN (1:1, v/v). Analytes were then eluted with 1 mL of 5% NH_4_OH in CH3OH:CH3CN (1:1, v/v). The elute was evaporated to dryness (40 °C) and then reconstituted in the HPLC solution for analysis (70 μL, 80% H_2_O in CH3CN) [24,25]. For single brain regions, due to the low tissue amount (between 10 and 30 mg), the above procedure was adapted accordingly. In this case, the samples were homogenized using 150 μL of pure methanol and then extracted in each step with a solvent volume that was half of that necessary for the samples containing 100 mg of tissue.

### 2.5 Sample collection

#### 2.5.1 Postnatal mice

C57BL/6 N male mice were generated using breeders originally obtained from Charles River Labs (West Sacramento, CA). Mice were weaned from dams at postnatal day (PND) 21 and group-housed in same-sex groups of 3−4 per cage. Mice were kept on a 12-h light, 12-h dark cycle and fed rodent chow (Lab Diet 5001; Purina USA) and water ad libitum. At 3 weeks of age, male mice were sacrificed under deep anesthesia with isoflurane (5%) followed by cervical dislocation and decapitation. Whole brains were rapidly dissected and then flash-frozen in 2-methylbutane (Fisher chemical, USA) over dry ice and in optimal cutting temperature (OCT) compound (Tissue-Tek) at − 80 °C until further use. Care and treatment of animals was performed in compliance with guidelines from and approved by the University of California Riverside Institutional Animal Care and Use Committee (AUP#20170026 and 20200018).

#### 2.5.2 Adult (15−16 Week old) mice

C57BL/6 N male mice were from Janvier (Le Genest-Saint-Isle, France). MCT8 and OATP1C1 dko mice (C57BL/6) were generated by Cyagen Biosciences Inc (Santa Clara, CA, USA) using CRISPR/Cas9-mediated genome engineering. The mice were engineered to lack the MCT8 coding exons 3−4 and the OATP1C1 coding exons 3−5. Successful MCT8 target gene deletion was confirmed using PCR analysis using the primers 5’-GAACAGCTCAGCCTTCCAAG-3’, 5’ -TGGAGTGGTTAGG-CAAGAGG-3’ and 5’-CCAAGTCCTCAGAGCTCCAA-3’ to yield 175 bp (WT) and 283 bp (KO), respectively. OATP1c1 deletion was confirmed using the primers 5’-GTTCCTCCCAAGACCACTCA-3’, 5’-AGT-CACGGTGCTCTTCAGAT-3’ and 5’-GGCCTATCCCTGTATGCACT-3’ to yield 420 bp (WT) or 240 bp (KO) respectively. All mice were group housed on a 12:12-h light-dark cycle at 22 °C with free access to chow diet and water. Mice were sacrificed by cervical dislocation and whole brains were rapidly dissected and snap frozen into liquid nitrogen and kept at − 80 ° C until analysis.

For different brain region analysis. C57BL/6 J (15−18 week old males) were from Janvier (Le Genest-Saint-Isle, France). Mice were group housed on a 12:12-h light-dark cycle at 22 °C with free access to rodent chow diet (Altromin, #1314) and water. Mice were sacrificed by cervical dislocation and brains were rapidly removed. Brain areas were micro-dissected and snap-frozen in liquid nitrogen and then kept at − 80 °C until analysis. All studies were performed according to the guidelines of the Institutional Animal Care and Use Committee of the State of Bavaria.

### 2.6 Method validation

The methodology was validated using 70−100 mg of mouse brain. The quantification limit (lower limit of quantitation, LOQ) was determined by spiking serially diluted calibrators containing the THs into the brain extract of MCT8/Oatp1c1 dko mice. The LOQ was defined as the lowest amount that gave a signal to noise ratio above 10.

The extraction efficacy (Table 1) was calculated by comparing the peak area of seven THs in spiked samples before (*n* = *3*) and after (*n* = *3*) extraction. This experiment was conducted by spiking a concentration of 8 pg/mg into brain extract of wild-type animals. The spike experiments were executed for T4, T3, rT3, 3,3’-T2, T1AM using the corresponding labeled internal standards. In this way the possible interferences due to the endogenous quantity of such THs were excluded. Currently, there are no commercially available labelled analogues for T1 and 3,5-T2, thus in that case the experiments were conducted using the native standards. Indeed, we considered the possible interferences due to the endogenous concentration of such metabolites very limited, since no T1 or 3,5-T2 was detected with our methodology. By comparing the peak areas of the standards spiked in the sample after the extraction (*n* = *3*) with those in neat solvent, the matrix effects, (expressed as ion suppression in Table 1) were also calculated.

The intra-day precision and accuracy of the method were assessed by preparing and measuring three different quality control samples (QC) on a single day. The quality control samples, for the intra-day measurement, were prepared by spiking standard solutions at different concentration (0.5 pg/μL, 5 pg/μL and 50 pg/μL) in the extract corresponding to a final concentration of 0.5 pg/mg, 5 pg/mg, 50 pg/mg in brain sample after subtracting for the endogenous amount. For the lowest level (0.5 pg/mg) the brain extract of MCT8/Oatp1c1 dko mice were used. In this way the possible interferences due to the endogenous thyroid hormone amount were limited. For the experiment at 5 and 50 pg/mg the brain of wild-type animals was employed. The inter-day precision and accuracy were tested by measuring five QC samples prepared on five different days. The inter-day precision was calculated only for two different concentration levels (5 and 50 pg/mg).

For single mouse brain regions the methodology was partially validated by determining the inter-day and the intra-day precision and accuracy only. For this validation about 15 mg of the entire mouse brain was used as surrogate. The quality control samples were prepared by spiking standard solutions at 1 pg/μL and 10 pg/μL into brain extract (15−18 mg). Taking into account the sample amount a final concentration of about 4 pg/mg and 40 pg/mg was expected after subtracting for the endogenous quantity. We considered less important to investigate the highest part of the calibration curve given the results obtained with the same experiment when the whole brain was used. The intra-day precision and accuracy of the method were assessed by preparing and measuring three different quality control samples (QC) on a single day. The inter-day precision and accuracy, were tested by measuring six QC samples prepared on six different days for the low level (4 pg/mg) and three QC samples prepared and measured on three different days for the high level (40 pg/mg). All quality control samples were prepared and treated in the same way as tissue samples and the standard solutions for spiking experiments were added immediately after sample homogenization.

Before sample analysis, QC samples were injected to test the good-ness of the calibration curve while for the instrumental quality control, regular injections of blanks and solution for the tuning of the instrument were performed. The analyte identification was based on the retention times compared with quantification standards and *m/z* ratios of the selected ions (Table S1). Moreover, we monitored the overall recovery of the quantification standards. The stability of the standards in solution as well as in tissue extract have been reported previously [24].

### 2.7 Statistical analysis

The concentrations were expressed as mean ± SD of at least three replicates. The concentrations in tissue are given per milligram of brain wet weight. Data acquisition, linearity of the standard curve and quantification of the samples were performed using Agilent MassHunter Workstation software.

## 3 Results and discussion

### 3.1 Method development

Initially, the clean-up procedure already developed in our group for the quantification of THs in tissues, was tested [25]. For this study, 70-100 mg of C57BL/6 N mouse brain were used. However, when the extracted THs were analyzed, a change in retention time was observed. This shift, likely due to the high lipid content of the brain, increased with the number of injections, and the peaks became even wider at every injection. This required several washings of the column before the initial conditions were restored. Consequently, a washing step with a mixture of hexane/chloroform was added to our procedure that prohibited further displacement or broadening of the peaks. The flow chart of the new protocol for the extraction of THs in mouse brain is shown in the supplementary material, Fig. S1. For the quantification of THs in single brain regions the same protocol was used but with a lower extraction volume.

Previously, we used a nano-aquity UPLC coupled with a Q-Tof mass detector for TH quantification in several different tissue [24,25,31,32]. However, when this instrumentation was applied for the measurement of THs in MCT8/Oatp1c1 dko mice, only T3 was quantified [33]. For this reason an UPLC-system interfaced with a triple-quadrupole (QQQ) mass detector was used for this study. The same system has been employed recently by our group for measuring thyroid hormones in breast milk [26]. In fact, the amount of THs in such a matrix is quite low and a very sensitive instrumentation is necessary. Therefore, the instrumentation parameters developed for the analysis of THs in breast milk were compared with those previously developed for the Q-Tof mass detector. With the UPLC-QQQ system, it was possible to lower the instrumental limit of detection by about ten times compared with the previous results obtained with the Q-Tof using standard solutions. Thus, such instrumentation and conditions were used for the quantification of THs in mouse brain and in single brain regions. The tandem mass spectrometer was operated under multiple reaction monitoring mode (MRM) and the fragmentations found are in line with those reported previously by others [22,34].

### 3.2 Validation study

The method was validated for mouse whole brain taking into account: accuracy, precision, limit of quantification, matrix effect, overall process efficacy and samples stability. For brain regions a partial validation was performed in which accuracy and precision were evaluated. Unfortunately, it was not possible to carry out a complete validation of the bioanalytical method as recommended by the FDA and/or EMA guidelines. The tissues of mice, particularly those from genetically modified animals, are not readily available. However, the information we provided for the validation of the method is in line with that reported by others on similar biological samples [21,22,34].

#### 3.2.1 Linearity

Calibration curves for all THs were made in neat solvents. Eight calibration standards, (0.35, 0.5, 1, 5, 10, 50, 100 and 150 pg/μL) with 10 pg/μL of internal standards, were prepared in H_2_O:CH_3_CN (8:2, v/v) containing 0.1% formic acid. In addition to these eight concentration levels, a blank and zero sample (solution containing internal standards only) were also included. The ratios of analyte peak area to internal standard peak area were plotted in the interval. Calibration curves obtained with a weighted linear regression (1/y), showed good linearity (r^2^ >0.996) in the tested concentration range. The linear regression equations for the seven thyroid hormones are reported in Table S2 of the supplementary material.

#### 3.2.2 Accuracy and precision

We used intra-day variation and inter-day variation to evaluate the precision of this method. We expressed the results in relative standard deviation (RSD %). As presented in Table 1, the intra-day and inter-day precision for all analytes, when the entire brain was used, is less than 15% except 3,5-T2 whose values are around 20% for the intra-day precision. For the single brain regions (Table 2) a relative standard deviation between 0.5% and 20.8% was reported. Despite in some cases the intra-day precision is higher than 15%, all the results obtained with the inter-day experiments showed a RSD close or lower such value.

We assessed the intra-day and the inter-day accuracy of this method by spike-recovery experiments. The results for the entire brain gave values between 77.4% and 109%. The low value obtained for T1 (77.4%) at 0.5 pg/mg can be explained by the fact that this experiment was conducted at a concentration slightly below the LOQ for such metabolite. For the single brain regions, a range between 80.2% and 128.6% was obtained. The high accuracy value of T4, after spiking 4 pg/mg standard solution in the sample, is probably due the interference of its endogenous concentration that is around 3 pg/mg. However, when this experiment was performed to calculate the inter-day accuracy a value of 103.8% was found. We considered that the optimized method had good precision and accuracy for the quantification of THs in the whole brain and an acceptable precision and accuracy for the measurement of THs in the different brain regions studied.

#### 3.2.3 Limit of quantification

**Fig. 2:**
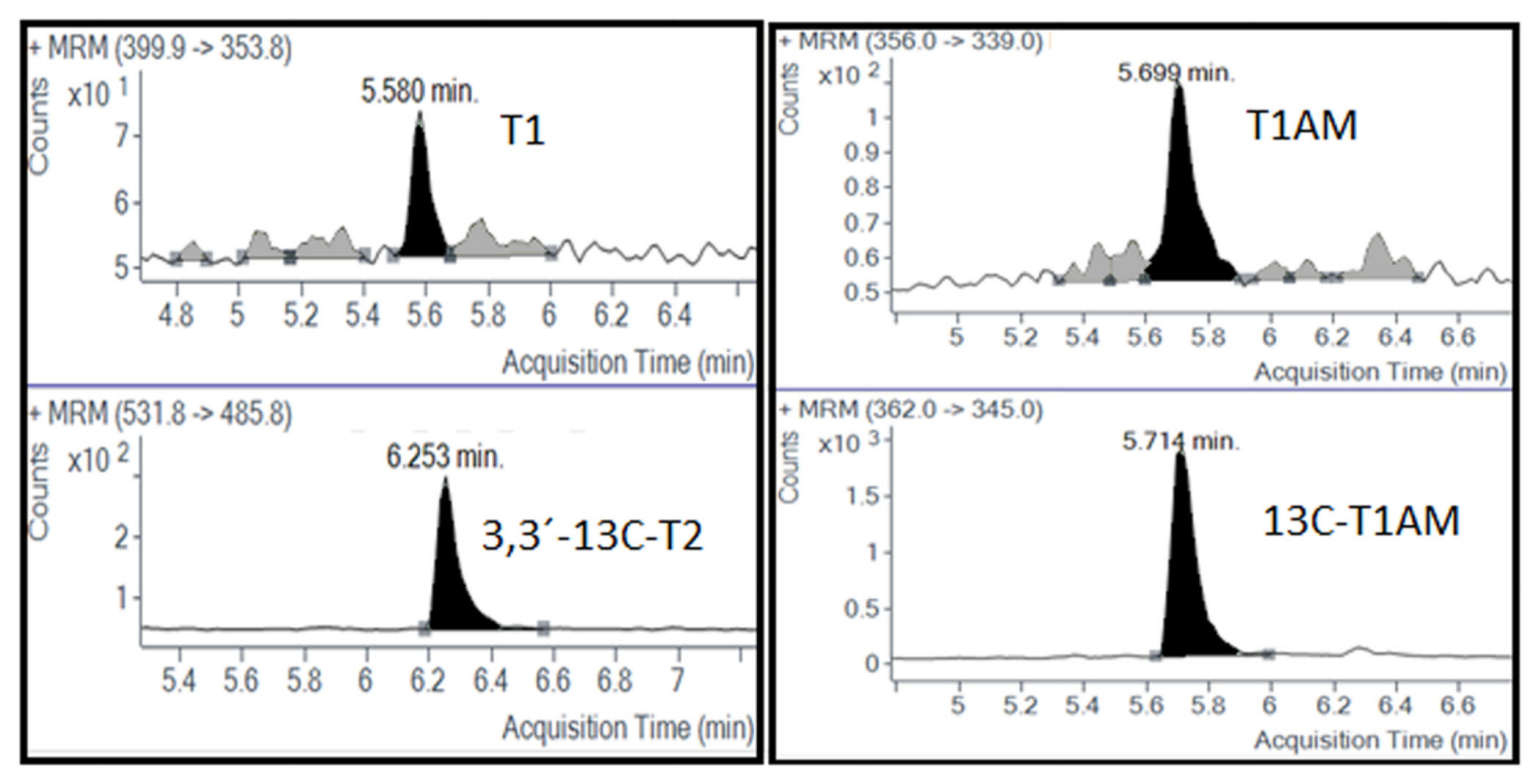
Chromatograms of T1 and T1AM with their corresponding internal standards, after spiking 0.66 and 0.50 pg/mg respectively into dko mouse brain extract.

As described above, the limit of quantification (LOQ) was determined by spiking serially diluted calibrators containing the THs into the brain extract of MCT8/Oatp1c1 dko mice. The dko mice were used to reduce the endogenous interferences as much as possible. In fact, in this tissue only a very low level of T3 and T4 was found (see next section) and no other TH metabolites were detected. Therefore, it could be considered a sort of blank matrix for rT3, 3,3’-T2, 3,5-T2, T1 and T1AM. As reported in Table 2, the LOQ for all THs is between 0.08 and 0.2 pg/mg except for T1 and T1AM which gave a value of 0.6 pg/mg and 0.35 pg/mg respectively. In this region of the chromatogram (Fig. 2), the samples are dirtier compared to the rest and this can explain the higher LOQ value.

#### 3.2.4 Process efficacy and matrix effect

In general, all THs showed a quite good extraction efficacy (>70%) except for T4, rT3 and T1AM whose values were around 50%. We considered the results obtained acceptable taking into account the number of steps necessary for sample purification. The matrix effect was approximately 50% for all THs with an overall process efficacy between 30% and 35%. We did not further dissect this low matrix effects by our extraction protocol, as both the accuracy and precision were within an acceptable range. Taking into account the results obtained with these experiments, the following threshold was defined: compared with neat analytical standards, samples with an intensity lower than 30% on peak area for internals standards were discarded, and if possible reprocessed.

#### 3.2.5 Sample stability

The stability of the calibration solutions, prepared and stored as described before, was reported in our earlier publication [24]. The possible degradation of internal standards in the biological matrix was also investigated in that publication using liver extracts and later in human placenta extracts including those subjected to freeze-thaw cycles [24,25]. Given the results obtained previously, we found it unnecessary to repeat the same experiments also in the mouse brain.

### 3.3 Quantification of THs in mouse brain

**Fig. 3:**
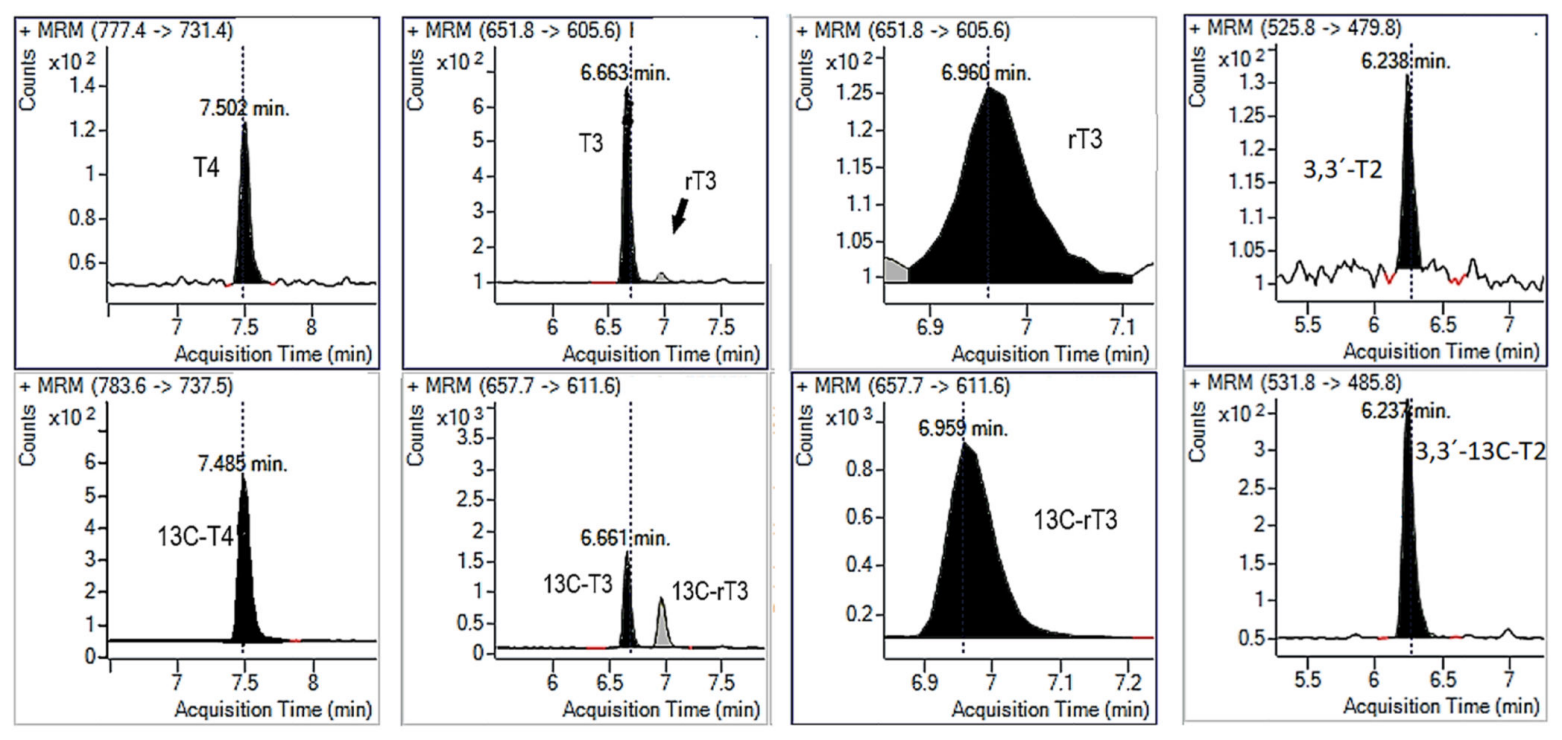
Representative chromatograms of THs detected in wild-type mouse brain tissue. The concentration found for this specific sample (108.9 mg) was: 1.78 ± 0.013 pg/mg for T3, 3.66 ± 0.192 pg/mg for T4, 0.213 ± 0.027 pg/mg for 3,3’-T2 and 0.209 ± 0.022 pg/mg for rT3.

The methodology was applied first to the measurement of mouse brain samples from one month old C57BL/6 N mice (*n* = *4*), using about 100 mg of tissue. Table 3 shows that in all samples tested we could measure T4, T3, rT3 and 3,3’-T2, although in one case the rT3 value was slightly below the LOQ. The concentration found for T4 and T3 was around 2-3 pg/mg while for rT3 and 3,3’-T2 a ten times lower level was detected. The concentration detected were quite similar between juvenile and adult brain samples. When about 400 mg of sample was used (n = 2, one male and one female mouse), no other THs were detected (data not shown). Fig. 3 shows the chromatograms of the detected THs compared to their internal standards measured in about 100 mg of brain sample from wild-type animal.

In the study of Manni and others, the authors were able to measure not only T4 and T3 but also T1AM in mouse brain [30]. Unfortunately, we could not detect such metabolite. This could perhaps be due to the fact that the authors used a different clean-up methodology and/or a different mouse strain (CD1 strain). However, it is important to note that with our protocol, it was possible to quantify for the first time rT3 and 3, 3’-T2. As far as we know, this is the first report in which the level of these metabolites were measured in mouse brain using the LC-MS/MS technique. Furthermore, compared to other LC-MS/MS methodologies, we used only about 100 mg of tissue amount out of the 400−500 mg previously described. To the best of our knowledge concentration levels of rT3 and 3,3’-T2 in rodents brain have been only reported by using RIA. Indeed, Pinna et al., using such technique were able to determine the concentration of seven TH metabolites in several rat brain regions. According to this report, rT3 and 3,3’-T2 were detected in almost all regions with concentrations that were approximately 10- to 20-fold lower than those of T3 [16]. In our study, rT3 and 3,3’-T2 concentrations were 7−16-times lower than T3 in one month-old mice. Thus, our LC-MS/MS data are consistent with RIA values reported earlier. Similarly, the levels of T3 and T4 in the brain quantified by our methodology were in agreement with previous reports in which these THs were analyzed with LC-MS/MS technique [22,30].

Subsequently, we assessed whether THs could be detected in different brain regions which have more limited quantity of tissue. Five different regions from male C57BL/6 *J* mice (*n = 5*) were tested: hypothalamus, hippocampus, prefrontal cortex, brainstem and cortex. In all regions T4 and T3 were quantified (Table 4). We measured a concentration between 1.99 pg/mg and 6.08 pg/mg for T4 and a value between 2.14 pg/mg and 2.73 pg/mg for T3. According to these data, the T4 value seems to vary more across the different brain regions than T3 concentration, which appears to be more constant. T4 and T3 in the hypothalamus, hippocampus and brainstem of rats were previously measured by others using RIA [14–16]. Those values were also reported for comparison in Table S3 of the supplementary material. In general, the values we report for the hypothalamus and brainstem are consistent with the ones reported previously, while, higher levels are found for the hippocampus, compared to those already published. No other TH metabolites were quantified in these brain regions, even if rT3 and 3,3’-T2 were both detected in all prefrontal cortex samples.

### 3.3.1 Preclinical application

Finally, we aimed to test if our methodology could detect predictably reduced levels of THs by analyzing brain samples from 15 to 16 week old MCT8/ OATP1C1 dko mice (*n = 6*) (Table 3). As expected, T3 and T4 levels were about 7-times lower in MCT8/ OATP1C1 dko brains than in brains from wild-type mice. No other TH metabolites were detected in the dko animals. In previous studies, where the levels of THs were measured by RIA on MCT8/ OATP1C1 dko mice, a similar trend was observed [12]. In the Fig. S2 of the supplementary material, is reported the chromatogram about the analysis of one dko mouse brain sample where the value found for T4 and T3 was close to the LOQ. These results prove that with our methodology we are able to detect central hypothyroidism of this dko mouse model. Furthermore, our results support previous reports and underscore the importance of TH transporters for proper TH homeostasis. Due to the low amount of T3 and T4 detected, no further test employing distinct mouse brain regions was conducted on MCT8/ OATP1C1 dko mice.

## 4 Conclusion

Here, we report validation of the LC-MS/MS method for the quantification of THs in mouse whole brains, in five distinct brain regions and its application in MCT8/ OATP1C1 dKO mouse brain. When using about 100 mg of C57BL/6 N whole brain sample four different THs were found: T4, T3, and, for the first time, rT3 and 3,3’-T2. When the methodology was applied to brain regions (C57BL/6 *J* mouse), despite the low tissue amount, T4 and T3 were consistently quantified across all regions. These results make our procedure one of the most sensitive thus far published for the detection of thyroid hormones in juvenile and adult mouse brain using LC-MS/MS. Accordingly, we believe that our methodology is a useful novel tool to dissect the impact of TH on the brain as well as specific brain regions of mice. In addition, our method can be used to detect the hypothyroid effects of endocrine-disrupting chemicals on the developing brain as shown by Kozlova et al. [35]. Finally, our methodology is particularly useful for the study of selective TH BBB transport of preclinical murine models for the AHDS, evidenced by our finding of profoundly diminished TH levels in brains of MCT8/OATP1C1 dKO mice.

## Supplementary Material

Suppl. Fig. 1

## Figures and Tables

**Table 1:** Method evaluation parameters for analyzing THs in mouse whole brain.

	T4	T3	rT3	3,3-T2	3,5-T2	T1	T1AM
LOQ (pg/mg)	0.2	0.08	0.15	0.18	0.21	0.6	0.35
Extraction efficacy (%)	50.2 ± 4.1	71.4 ± 5.0	56.1 ± 6.6	71.7 ± 0.6	82.2 ± 5.1	83.2 ± 3.7	55.2 ± 10.9
Ion suppression (%)	-47.3 ± 3.7	-52.3 ± 4.0	-44.6 ± 1.6	-43.9 ± 2.9	-44.0 ± 9.1	-41.8 ± 6.8	-43.4 ± 8.6
Intra-day accuracy (%), *n* = *5*							
5 (pg/mg)	104.8^a^	100.3	94.3	95.9	87.3	98.2	99.0^a^
50 (pg/mg)	91.7	99.6	92.7	100.3	98.5	102.1	99.5^a^
Intra-day precision (%), *n* = *5*							
5 (pg/mg)	14.02^a^	10.93	5.97	6.13	18.13	9.69	9.92^a^
50 (pg/mg)	11.71	4.20	8.76	5.19	20.07	11.76	5.45^a^
Inter-day accuracy (%), *n* = 3							
0.5 (pg/mg)	114.78	119.20	98.23	84.91	97.9	75.04	109.4
5 (pg/mg)	102.8	91.2	95.2	93.4	94.4	97.9	87.0
50 (pg/mg)	104.2	102.3	99.1	102.9	108.9	106.0	102.6
Inter-day precision (%), *n* = *3*							
0.5 (pg/mg)	17.96	6.55	9.83	12.10	13.85	14.51	0.4
5 (pg/mg)	17.37	9.68	5.81	5.13	8.49	11.65	6.33
50 (pg/mg)	2.44	3.63	2.81	4.11	6.93	6.89	2.68

a The experiment was done in 4 replicates

**Table 2:** Intra-day and inter-day accuracy and precision for analyzing THs in mouse brain regions.

	T4	T3	rT3	3,3’-T2	3,5-T2	T1	T1AM
Intra-day accuracy (%), *n* = 6							
4 (pg/mg)	128.6	101.5	97.5	92.3	104.5	120.7	112.6
40 (pg/mg)^a^	105.3	102.5	94.4	86.5	98.5	97.9	98.4
Intra-day precision (%), *n* = *6*							
4 (pg/mg)	16.71	9.82	5.97	15.51	11.87	10.7	20.86
40 (pg/mg)^a^	6.23	3.01	0.55	9.11	5.97	8.23	12.25
Inter-day accuracy (%), *n* = *3*							
4 (pg/mg)	103.8	110.4	91.9	88.5	123.5	120.4	101.2
40 (pg/mg)	107.3	96.4	93.8	80.2	100.3	89.9	108.1
Inter-day precision (%), *n* = *3*							
4 (pg/mg)	13.30	5.64	7.51	11.42	15.05	6.75	4.34
40 (pg/mg)	3.95	6.48	3.27	15.6	2.53	5.18	4.96

a The experiment was done in triplicates.

**Table 3:** Determination of TH concentration in male mouse brain samples.

	T4 (pg/mg)	T3 (pg/mg)	rT3 (pg/mg)	3,3’-T2 (pg/mg)
1 month old (juvenile)				
C57Bl/6 N (WT, *n* = *4*)	3.17 ± 0.90	2.42 ± 0.16	0.15 ± 0.04	0.35 ± 0.03
15-16 week old (adult)				
C57Bl/6 N (WT, *n* = *3*)	2.48 ± 0.62	2.13 ± 0.38	0.28 ± 0.08	0.6 ± 0.15
MCT8/Oatp1c1 dKO (*n* = *6*)	0.38 ± 0.31	0.29 ± 0.13	< LOQ	< LOQ

**Table 4:** Determination of TH concentration in specific brain regions of adult male C57Bl/6 J mice (*n* = *5*).

Brain region	T4 (pg/mg)	T3 (pg/mg)
Hypothalamus	3.44 ± 0.66	2.30 ± 0.18
Hippocampus	6.08 ± 2.19	2.50 ± 0.26
Prefrontal cortex	1.99 ± 0.55	2.14 ± 0.22
Cortex	2.62 ± 0.39	2.68 ± 0.24
Brainstem	4.70 ± 0.78	2.73 ± 0.62

## Data Availability

Data will be made available on request.

## References

[1] Lopez M, Alvarez CV, Nogueiras R, Dieguez C (2013). Energy balance regulation by thyroid hormones at central level. Trends Mol Med.

[2] Cheng S-Y, Leonard JL, Davis PJ (2010). Molecular aspects of thyroid hormone actions. Endocr Rev.

[3] Scanlan TS, Suchland KL, Hart ME, Chiellini G, Huang Y, Kruzich PJ, Frascarelli S, Crossley DA, Bunzow JR, Ronca-Testoni S, Lin ET (2004). 3-iodothyronamine is an endogenous and rapid-acting derivative of thyroid hormone. Nat Med.

[4] Piehl S, Hoefig CS, Scanlan TS, Kohrle J (2011). Thyronamines−past, present, and future. Endocr Rev.

[5] Ianculescu AG, Scanlan TS (2010). 3-Iodothyronamine (T(1)AM): a new chapter of thyroid hormone endocrinology?. Mol Biosyst.

[6] Glossmann HH, Lutz OMD (2017). Torpor: the rise and fall of 3-Monoiodothyronamine from brain to gut-from gut to brain?. Front Endocrinol.

[7] Scanlan ST (2009). Minireview: 3-Iodothyronamine (T1AM): a new player on the thyroid endocrine team?. Endocrinology.

[8] Homuth G, Lietzow J, Schanze N, Golchert J, Köhrle J (2020). Endocrine, metabolic and pharmacological effects of thyronamines (TAM), thyroacetic acids (TA) and thyroid hormone metabolites (THM) - evidence from in vitro, cellular, experimental animal and human studies. Exp Clin Endocrinol Diabetes.

[9] Stromme P, Groeneweg S, de Souza EC Lima, Zevenbergen C, Torgersbraten A, Holmgren A, Gurcan E, Meima ME, Peeters RP, Visser WE, Honeren Johansson L (2018). Mutated thyroid hormone transporter OATP1C1 associates with severe brain hypometabolism and juvenile neurodegeneration. Thyroid.

[10] Grijota-Martinez C, Barez-Lopez S, Gomez-Andres D, Guadano-Ferraz A (2020). MCT8 deficiency: the road to therapies for a rare disease. Front Neurosci.

[11] Schwartz CE, May MM, Carpenter NJ, Rogers RC, Martin J, Bialer MG, Ward J, Sanabria J, Marsa S, Lewis JA, Echeverri R (2005). Allan-herndon-dudley syndrome and the monocarboxylate transporter 8 (MCT8) gene. Am J Hum Genet.

[12] Mayerl S, Muller J, Bauer R, Richert S, Kassmann CM, Darras VM, Buder K, Boelen A, Visser TJ, Heuer H (2014). Transporters MCT8 and OATP1C1 maintain murine brain thyroid hormone homeostasis. J Clin Invest.

[13] de Escobar G Morreale, Pastor R, Obregon MJ, del Rey F Escobar (1985). Effects of maternal hypothyroidism on the weight and thyroid hormone content of rat embryonic tissues, before and after onset of fetal thyroid function. Endocrinology.

[14] Campos-Barros A, Meinhold H, Walzog B, Behne D (1997). Effects of selenium and iodine deficiency on thyroid hormone concentrations on the central nervous system of the rat. Eur J Endocrinol.

[15] Pinna G, Hiedra L, Prengel H, Broedel O, Eravci M, Meinhold H, Baumgartner A (1999). Extraction and quantification of thyroid hormones in selected regions and subcellular fractions of the rat brain. Brain Res Protoc.

[16] Pinna G, Brödel O, Visser TJ, Jeitner A, Grau H, Eravci M, Meinhold H, Baumgartner A (2002). Concentrations of seven iodothyronine metabolites in brain regions and the liver of the adult rat. Endocrinology.

[17] Rathmann D, Rijntjes E, Lietzow J, Kohrle J (2015). Quantitative analysis of thyroid hormone metabolites in cell culture samples using LC-MS/MS. Eur Thyroid J.

[18] Soldin Steven J, Soukhova N, Janicic Natasa, Jonklaas Jacqueline, Soldin Offie P (2005). The measurement of free thyroxine by isotope dilution tandem mass spectrometry. Clin Chim Acta.

[19] Gounden V, Jonldaas J, Soldin SJ (2014). A pilot study: subclinical hypothyroidism and free thyroid hormone measurement by immunoassay and mass spectrometry. Clin Chim Acta.

[20] Grova N, Wang X, Hardy EM, Palazzi P, Chata C, Appenzeller BMR Ultra performance liquid chromatography - tandem mass spectrometer method applied to the analysis of both thyroid and steroid hormones in human hair. J Chromatogr A.

[21] Saba A, Donzelli R, Colligiani D, Raffaelli A, Nannipieri M, Kusmic C, Dos Remedios CG, Simonides WS, Lervasi G, Zucchi R (2014). Quantification of thyroxine and 3,5,3-triiodo-thyronine in human and animal hearts by a novel liquid chromatography-tandem mass spectrometry method. Horm Metab Res.

[22] Kunisue JWFT, Kannan K (2011). Determination of six thyroid hormones in the brain and thyroid gland using isotope-dilution liquid chromatography/tandem mass spectrometry. Anal Chem.

[23] Ackermans MT, Kettelarij-Haas Y, Boelen A, Endert E (2012). Determination of thyroid hormones and their metabolites in tissue using SPE UPLC-tandem MS. Biomed Chromatogr: BMC.

[24] De Angelis M, Giesert F, Finan B, Clemmensen C, Muller TD, Vogt-Weisenhorn D, Tschop MH, Schramm KW (2016). Determination of thyroid hormones in mouse tissues by isotope-dilution microflow liquid chromatography-mass spectrometry method. J Chromatogr B Anal Technol Biomed Life Sci.

[25] Li ZM, Giesert F, Vogt-Weisenhorn D, Main KM, Skakkebaek NE, Kiviranta H, Toppari J, Feldt-Rasmussen U, Shen HQ, Schramm KW, De Angelis M (2018). Determination of thyroid hormones in placenta using isotope-dilution liquid chromatography quadrupole time-of-flight mass spectrometry. J Chromatogr A.

[26] Li ZM, Albrecht M, Fromme H, Schramm KW, De Angelis M (2020). Persistent organic pollutants in human breast milk and associations with maternal thyroid hormone homeostasis. Environ Sci Technol.

[27] Noyes PD, Lema SC, Roberts SC, Cooper EM, Stapleton HM (2014). Rapid method for the measurement of circulating thyroid hormones in low volumes of teleost fish plasma by LC-ESI/MS/MS. Anal Bioanal Chem.

[28] Chen X, Walter KM, Miller GW, Lein PJ, Puschner B (2018). Simultaneous quantification of T4, T3, rT3, 3,5-T2 and 3,3’-T2 in larval zebrafish (Danio rerio) as a model to study exposure to polychlorinated biphenyls. Biomed Chromatogr: BMC.

[29] Saba A, Chiellini G, Frascarelli S, Marchini M, Ghelardoni S, Raffaelli A, Tonacchera M, Vitti P, Scanlan TS, Zucchi R (2010). Tissue distribution and cardiac metabolism of 3-Iodothyronamine. Endocrinology.

[30] Manni ME, De Siena G, Saba A, Marchini M, Landucci E, Gerace E, Zazzeri M, Musilli C, Pellegrini-Giampietro D, Matucci R, Zucchi R (2013). Pharmacological effects of 3-iodothyronamine (T1AM) in mice include facilitation of memory acquisition and retention and reduction of pain threshold. Br J Pharm.

[31] Li ZM, Hernandez-Moreno D, Main KM, Skakkebaek NE, Kiviranta H, Toppari J, Feldt-Rasmussen U, Shen HQ, Schramm KW, De Angelis M (2018). Association of in utero persistent organic pollutant exposure with placental thyroid hormones. Endocrinology.

[32] Li ZM, Benker B, Bao Q, Henkelmann B, Corsten C, Michalke B, Pauluschke-Frohlich J, Flisikowski K, Schramm KW, De Angelis M (2020). Placental distribution of endogenous and exogenous substances: a pilot study utilizing cryo-sampled specimen off delivery room. Placenta.

[33] Sundaram SM, Arrulo Pereira A, Muller-Fielitz H, Kopke H, De Angelis M, Muller TD, Heuer H, Korbelin J, Krohn M, Mittag J, Nogueiras R (2022). Gene therapy targeting the blood-brain barrier improves neurological symptoms in a model of genetic MCT8 deficiency. Brain.

[34] Hansen M, Luong X, Sedlak DL, Helbing CC, Hayes T (2016). Quantification of 11 thyroid hormones and associated metabolites in blood using isotope-dilution liquid chromatography tandem mass spectrometry. Anal Bioanal Chem.

[35] Kozlova EV, De Angelis M, Denys ME, Schramm K-W, Curras-Collazo MC (2021). Sex and time dependent effects of developmental PBDE on hypothalamo-pituitary-thyroid axis are partially restored by maternal levothyroxine supplementation in C57BL/6 mice. Organohalogen Compounds.

