# Supplementary material for: Development and validation of an LC-MS/MS methodology for the quantification of thyroid hormones in dko MCT8/OATP1C1 mouse brain
Source: J Pharm Biomed Anal. Author manuscript; Available in PMC 2022 Oct 26. (PMC7613747; doi:10.1016/j.jpba.2022.115038)
Supplement: Suppl. Fig. 1 [file EMS155139-supplement-Suppl__Fig__1.docx]

**Development and validation of an LC-MS/MS methodology for the quantification of thyroid hormones in dko MCT8/OATP1C1 mouse brain**

Meri De Angelis^1,^*, Gandhari Maity-Kumar^2^, Sonja C. Schriever^2,3,4^, Elena V. Kozlova^5^, Timo D. Müller^2,3^, Paul T. Pfluger^2,3,4,6^, Margarita C. Curras-Collazo^5^, Karl-Werner Schramm^1,7^

^1^Helmholtz Zentrum München-German Research Center for Environmental Health (GmbH), Molecular EXposomics, Ingolstädter Landstr. 1, Neuherberg, Germany

^2^Institute for Diabetes and Obesity, Helmholtz Diabetes Center at Helmholtz Zentrum München, Neuherberg, Germany

^3^German Center for Diabetes Research (DZD), Neuherberg, Germany

^4^Research Unit Neurobiology of Diabetes, Helmholtz Zentrum München, Neuherberg, Germany

^5^Department of Molecular, Cell and Systems Biology, University of California, Riverside, USA

^6^TUM School of Medicine, Neurobiology of Diabetes, Technical University Munich, Germany

^7^Department für Biowissenschaftliche Grundlagen, Technische Universität München, Weihenstephaner Steig 23, Freising, Germany

**Correspondence:**

*Meri De Angelis, Helmholtz Zentrum München-German Research Center for Environmental Health (GmbH), Molecular EXposomics, Ingolstädter Landstr. 1, 85764 Neuherberg, Germany.

E-mail:[meri.deangelis@helmholtz-muenchen.de](mailto:meri.deangelis@helmholtz-muenchen.de)

Tel. +49 89 3187 2932

**Table S1** Optimized MS/MS parameters for THs. For each compound, ion-transitions are shown as m/z for the parent ion and two product ions (for quantification (q) and confirmation (c)). Compound optimized values for retention time (t_R_), fragmentor (F), collision energy (CE), collision acceleration voltage (CAV) and dwell times

| Compound | t_R_ (min) | Parent ion (m/z) | Product ions (m/z) | F (V) | CE (V) | CAV (V) | Dwell  (msec) |
| --- | --- | --- | --- | --- | --- | --- | --- |
| Target compounds |  | | | | | | |
| T4 | 7.49 | 777.4 | 731.4 (q) | 140 | 25 | 1 | 50 |
|  |  |  | 633.4 (c) | 140 | 25 | 1 | 50 |
| T3 | 6.66 | 651.8 | 605.6 (q) | 120 | 25 | 1 | 50 |
|  |  |  | 507.6 (c) | 120 | 25 | 1 | 50 |
| rT3 | 6.96 | 651.8 | 605.6 (q) | 120 | 25 | 1 | 50 |
|  |  |  | 507.6 (c) | 120 | 25 | 1 | 50 |
| 3,3’-T2 | 6.24 | 525.8 | 479.8 (q) | 90 | 15 | 2 | 50 |
|  |  |  | 381.8 (c) | 90 | 15 | 2 | 50 |
| 3,5-T2 | 5.91 | 525.8 | 479.8 (q) | 90 | 15 | 2 | 50 |
|  |  |  | 381.8 (c) | 90 | 15 | 2 | 50 |
| T1 | 5.58 | 399.9 | 353.8 (q) | 90 | 12 | 1 | 50 |
|  |  |  | 256 (c) | 90 | 15 | 1 | 50 |
| T1AM | 5.73 | 356 | 339 (q) | 80 | 18 | 1 | 50 |
|  |  |  | 212 (c) | 80 | 18 | 1 | 50 |
| Internal standards |  | | | | | | |
| ^13^C_6_-T4 | 7.49 | 783.6 | 737.5 | 140 | 25 | 1 | 50 |
| ^13^C_6_-T3 | 6.66 | 657.7 | 611.6 | 120 | 25 | 1 | 50 |
| ^13^C_6_-rT3 | 6.96 | 657.7 | 611.6 | 120 | 25 | 1 | 50 |
| ^13^C_6_-3,3’-T2 | 6.24 | 531.8 | 485.8 | 100 | 25 | 2 | 50 |
| ^13^C_6_-T1AM | 5.73 | 362 | 345 | 104 | 12 | 1 | 50 |

100 mg mouse brain sample in 300 µL MeOH

**Homogenization & IS addition:**

- Ultrasonication, for 1 x 30s, under cooling with ice
- Internal standard addition: ^13^C_6_-T4, ^13^C_6_-T3, ^13^C_6_-rT3, ^13^C_6_-T2, ^13^C_6_-T1AM + 600 µL CHCl_3_

**Extraction THs from tissue:**

- Centrifugation, 10 min, 3000 g, 4°C
- Separation of the supernatant
- Repetition extraction 300 µl MeOH + 600 µL CHCl_3_

**Liquid-liquid extraction:**

- Addition to the supernatant: 0.05% Ca_2_Cl in H_2_O (2x0.8 mL)
- Centrifugation 10 min, 3000 g, 4°C
- Separation of the upper phase
- Washing of the inorganic phase: 2x2mL hexane:CHCl_3_ (9:1)
- Addition to the inorganic phase of 1ml pure water+ H_3_PO_4_ (1%, v/v)

**Bond Elut Plexa PCX, solid phase extraction:**

- Conditioning: 2 mL MeOH, 2 mL H_2_O
- Sample loading followed by washing: 2 mL 1% FA in H_2_O, 2 mL MeOH:CH_3_CN (1:1) & elution: 1 mL 5% NH_3_ in MeOH:CH_3_CN (1:1)

Evaporation to dryness, 40° C

**UPLC-MS/MS analysis:**

- Re-dissolve in 70 µL of H_2_O:CH_3_CN (8:2) & Injection

**Fig. S1** Protocol of TH analysis in mouse brain

**Table S2** Linear regression equations (y= analyte area/IS area; x= analyte concentration/IS concentration) for the seven THs

| Thyroid hormones | Equation | Correlation coefficient (r^2^) |
| --- | --- | --- |
| T1 | y= 1.123x + 0.009 | 0.9981 |
| T1AM | y=0.405x + 0.001 | 0.9998 |
| 3,5-T2 | y=3.191x + 0.006 | 0.9986 |
| 3,3´-T2 | y=1.932x + 0.012 | 0.9984 |
| T3 | y= 1.276x + 0.012 | 0.9992 |
| rT3 | y= 1.454x + 0.009 | 0.9969 |
| T4 | y=0.391x + 0.004 | 0.9998 |

**Table S3** Thyroid hormones concentration in rat brain regions reported previously

| Brain region | T4 | T3 |
| --- | --- | --- |
| Hypothalamus | 1.92 ± 0.20 pg/mg^a^  ̴5000 fmol/g^b^ | 1.55 ± 0.13 pg/mg^a^  ̴2500 fmol/g^b^ |
| Hippocampus | 3.26 ± 0.44 pg/mg^a^  ̴3000 fmol/g^b^ | 1.26 ± 0.14 pg/mg^a^  ̴1500 fmol/g^b^ |
| Brainstem | 4.49 ± 0.57 pg/mg^a^ | 2.03 ± 0.12 pg/mg^a^ |

^a^Campos-Barros *et al.* [1] (Wistar rats, two months old)

^b^ Pinna *et al.* [2] (Adult male euthyroid Sprague Dawley rats)

**
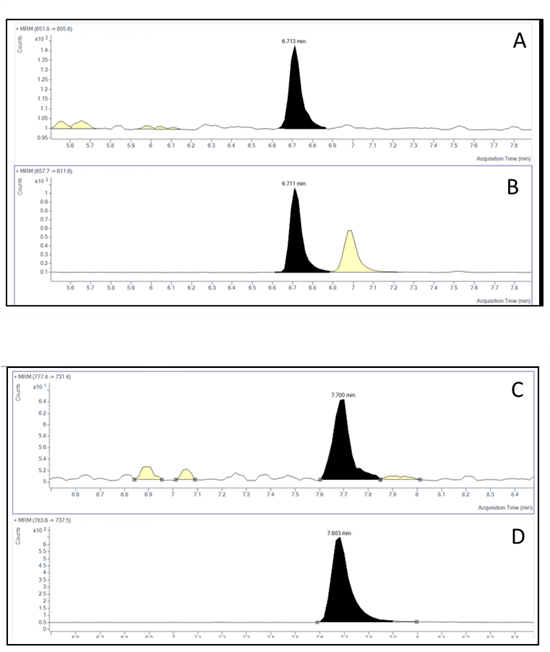
**

**Fig. S2** Representative chromatograms of THs detected in dko mouse brain tissue: (A) T3; (B) ^13^C_6_-T3 and ^13^C_6_-rT3; (C) T4; (D) ^13^C_6_-T4. The concentration found was 0.143 ± 0.008 pg/mg for T3 and 0.247±0.029 pg/mg for T4. The measurement was conducted using two different brain samples.

**References**

[1] A. Campos-Barros, H. Meinhold, B. Walzog, D. Behne, Effects of Selenium and Iodine Deficiency on Thyroid Hormone Concentrations on the Central Nervous System of the Rat, Eur J Endocrinol 136 (1997) 316-323.

[2] G. Pinna, O. Brödel, T.J. Visser, A. Jeitner, H. Grau, M. Eravci, H. Meinhold, A. Baumgartner, Concentrations of Seven Iodothyronine Metabolites in Brain Regions and the Liver of the Adult Rat, Endocrinology 143(5) (2002) 1789-1800.
